# Biowastes as Reinforcements for Sustainable PLA-Biobased Composites Designed for 3D Printing Applications: Structure–Rheology–Process–Properties Relationships

**DOI:** 10.3390/polym18010128

**Published:** 2025-12-31

**Authors:** Mohamed Ait Balla, Abderrahim Maazouz, Khalid Lamnawar, Fatima Ezzahra Arrakhiz

**Affiliations:** 1 Université de Lyon, INSA Lyon, Université Jean Monnet, CNRS UMR 5223, Ingénierie des Matériaux Polymères, F-69621 Villeurbanne Cédex, France; mohamed.ait-balla@insa-lyon.fr (M.A.B.); khalid.lamnawar@insa-lyon.fr (K.L.); 2 Laboratory of Materials, Signals, Systems and Physical Modeling, Faculty of Science, Ibn Zohr University, Dakhla City, B.P.8106, Agadir 80000, Morocco; f.arrakhiz@uiz.ac.ma; 3 Hassan II Academy of Science and Technology, Rabat 10100, Morocco

**Keywords:** poly (lactic acid), natural fibers, bio-composites, melt processing, solvent casting, morphological, thermal, mechanical, rheological properties, 3D printing

## Abstract

This work focused on the development of eco-friendly bio-composites based on polylactic acid (PLA) and sugarcane *bagasse* (SCB) as a natural fiber from Moroccan vegetable waste. First, the fiber surface was treated with an alkaline solution to remove non-cellulosic components. Then, the composite materials with various amounts of treated sugarcane *bagasse* (TSCB) were fabricated using two routes, melt processing and solvent casting. The primary objective was to achieve high fiber dispersion/distribution and homogeneous bio-composites. The dispersion properties were analyzed using scanning electron microscopy (SEM). Subsequently, the thermal, mechanical, and melt shear rheological properties of the obtained PLA-based bio-composites were investigated. Through a comparative approach between the dispersion state of fillers with extrusion/injection molding and solvent casting method, the work aimed to identify the most suitable processing route for producing PLA-based composites with optimal dispersion, improved thermal stability, and mechanical reinforcement. The results support the potential of TSCB fibers as an effective bio-based additive for PLA filament production, paving the way for the development of eco-friendly and high-performance materials designed for 3D printing applications. Since the solvent-based route did not allow further improvement and presents clear limitations for large-scale or industrial implementation, the transition toward 3D printing became a natural progression in this work. Material extrusion offers several decisive advantages, notably the ability to preserve the original morphology of the fibers due to the moderate thermo-mechanical stresses involved, and the possibility of manufacturing complex geometries that cannot be obtained through conventional injection molding. Although some printing defects may occur during layer deposition, the mechanical properties obtained through 3D printing remain promising and demonstrate the relevance of this approach.

## 1. Introduction

In a context where sustainable development has become a key priority in the field of polymer materials, there is a significant increase in the development of polymers derived from renewable resources [1,2,3]. The ecological benefit of these materials lies in their natural ability for degradation, as they can be directly broken down by soil microorganisms. Economically, applications such as food packaging show considerable potential for growth [4,5]. Poly (lactic acid) (PLA) is a biodegradable polymer synthesized from monomers obtained from renewable resources. It can be sourced from plant materials such as corn, beets, potatoes, and sugarcane *bagasse* [6]. This polymer has a wide range of applications and has garnered considerable attention from both the scientific and industrial sectors thanks to its biocompatibility and biodegradability [7,8].

Several recent works have highlighted the potential of lignocellulosic reinforcements for the development of sustainable polymer-based materials. Studies have shown that natural and chemically treated lignocellulosic fibers can improve the stability, interfacial activity, and overall performance of bio-composites [9,10,11]. Recent reviews have also underlined the sustainable nature and practical benefits of natural fibers [12], while nano-reinforcements such as nanocellulose or silica were shown to strengthen polymer matrices by enhancing stiffness and reinforcement efficiency [12,13]. Moreover, Qiao et al. reported that improved dispersion of cellulosic nano-fillers in PLA can enhance crystallization behavior and mechanical properties in melt-processed systems [1]. Together, these findings underline the key role of lignocellulosic fibers and nano-scale fillers in advancing high-performance, bio-based composite materials.

Several other studies have also focused on the blending of biodegradable polymers with natural fibers. This approach enables the examination of the dispersion state, morphological control, and mechanical properties. For instance, Mazzanti et al. [12] developed bio-composites using treated and untreated hemp fibers, employing an alkali treatment to enhance fiber-matrix adhesion and improve the mechanical properties of the resulting bio-composites, while also reducing the occurrence of fiber bundles. Khan et al. [13] examined the physical, mechanical, and thermal properties of hybrid composites made from polylactic acid (PLA) reinforced with bamboo (BF) and kenaf (KF) fibers, aiming to develop eco-friendly bio-composites. In another study, Khamakhem et al. [14] explored the impact of olive solid waste fillers on the morphological, mechanical, and rheological properties, analyzing their effects on the thermal stability, as well as the melt and crystallization behavior, both with and without the use of a compatibilizer.

Despite the many studies on the abovementioned topic, there is still a lack of comparative research on the state of fiber dispersion and its effect on the mechanical and thermal properties of bio-composites based on biodegradable polymers and natural fibers. Herein, we aim to fill this gap by conducting a comparative analysis of fiber dispersion and its impact on the mechanical and thermal properties, as well as carrying out a rheological study to optimize their injection molding and 3D printing processing parameters. Since bio-composites, particularly those based on PLA, are thermally sensitive, it is crucial to first study their rheological behavior to prevent degradation during processing at the melt state. This aspect, which is essential for improving the performance of PLA-based materials, has received very little coverage in literature.

Although sugarcane *bagasse* has been widely investigated in conventional polymer composites, its integration into advanced manufacturing technologies such as 3D printing remains underdeveloped. Three-dimensional printing manufacturing offers the potential for precision, minimal waste, and customizability, yet the integration of agro-waste fibers faces challenges, including poor dispersion, fiber–matrix incompatibility, and limited filament processability. Moreover, the influence of critical manufacturing parameters such as printing temperature, reinforcement content, and infill density on the structural integrity and overall performance of *bagasse*-reinforced composites has received little attention [15,16]. These combined limitations restrict the optimization and industrial adoption of sugarcane *bagasse* for engineering applications through additive manufacturing.

Finally, this study investigates the influence of two processing techniques—melt extrusion and solvent casting—on the morphological, thermal, mechanical, and rheological properties of PLA composites reinforced with sugarcane *bagasse* (SCB) fibers. Composites were prepared with varying fiber loadings (0, 5, 10, and 15 wt%) using residual *bagasse* as a sustainable filler. A comprehensive characterization was conducted to assess the crystallization behavior, structural integrity, and performance of the resulting materials, with a particular focus on fiber dispersion and its correlation with property enhancement. The study highlighted that solvent casting rendered possible the production of composites with a homogeneous fiber dispersion while preserving their initial structure. However, this non-destructive method presents ecological drawbacks and challenges for industrial-scale development. In contrast, the melt processing approach ensured good fiber dispersion despite some degree of fiber damage, which did not significantly compromise the desired final properties. Furthermore, this study explores the potential use of these fiber-reinforced composites for material extrusion (MEX)-3D printing applications. In this context, a comparative analysis of the mechanical properties between injection-molded and 3D-printed specimens was also conducted to assess the impact of processing methods on material performance, the aim being to assess the printability of PLA/SCB filaments and optimize printing conditions to produce printed components with consistent quality and optimized functional properties.

## 2. Materials and Methods

### 2.1. Materials

The polymer selected for this study was a commercial semi-crystalline polylactic acid (PLA, Ingeo 2003 D), containing 4.0% D-isomer lactide with an average molecular weight (Mw) of approximately 200,000 g·mol^−1^ and supplied by NatureWorks (Plymouth, MN, USA). Table 1 summarizes its main characteristics and properties. Sugarcane *bagasse* (SCB) residues collected from the Agadir region in Morocco were used as natural fiber reinforcement.

### 2.2. Fiber Extraction and Preparation

**Figure 1:**
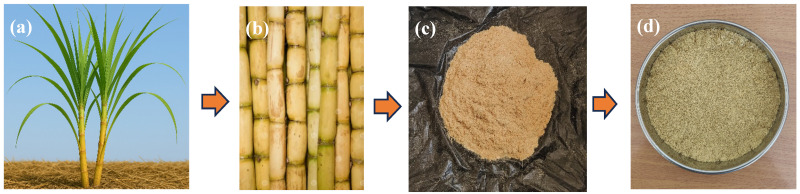
Extraction overview of sugarcane *bagasse* fibers from *bagasse* residue: (**a**) sugarcane *bagasse* plant, (**b**) sugarcane extracted from the original plant, (**c**) sugarcane *bagasse* fibers after grinding, and (**d**) sieved fibers with a mesh size ≤ 225 µm.

Raw sugarcane *bagasse* was collected from the Agadir region (Morocco) and initially washed with warm water to facilitate the separation of pulp from fibers. The recovered fibers were then thoroughly rinsed to eliminate any surface impurities, followed by drying in a hot-air oven at 80 °C for 12 h to remove residual moisture. Infrared and morphological analyses were carried out; the details are described in the Supplementary Materials. Once dried, the fibers were ground using a mechanical grinder (“Broy”) operating at 28,000 rpm with a 3200 W motor and a 1 kg capacity, in order to obtain a finer and more homogeneous size distribution. Fiber dimensions were assessed using optical microscopy, based on 150 individual measurements. The average fiber length and diameter were approximately 600 µm and 135 µm, respectively. The overall preparation steps are illustrated in Figure 1.

### 2.3. Chemical Treatment of Bagasse Fibers

Alkali treatment was carried out to partially eliminate non-cellulosic components and surface impurities, while also reducing the hydrophilicity of the lignocellulosic fibers. [17]. The SCB fibers were first ground and then immersed in a sodium hydroxide aqueous solution at a concentration of 2 wt% NaOH for approximately 2 h at room temperature (25 °C). This content was selected based on our previous study [9]. Following this treatment, the fibers were successively rinsed with distilled water and twice with a 2% acetic acid solution to neutralize any residual NaOH. A final series of distilled water rinses was then carried out to remove any remaining traces of the treatment reagents. Finally, the treated fibers were dried in an electric oven at 80 °C for 24 h. In the remainder of this article, we refer to the alkali-treated sugarcane *bagasse* fibers as (TSCB).

### 2.4. Processing and Preparation of the Bio-Composites

#### 2.4.1. Melt Extrusion

Prior to compounding, PLA pellets and fiber fillers were vacuum-dried at 70 °C and 80 °C, respectively, for 8 h to eliminate residual moisture. Composite preparation was carried out using a co-rotating conical twin-screw extruder (15 mL Micro-compounder, DSM Xplore, Geleen, The Netherlands) under a nitrogen atmosphere to limit polymer degradation during melt processing. For some reason, specific processing parameters were applied: 170 °C for 3 min at a rotor speed of 100 rpm. These parameters were determined in advance through rheological measurements and thermogravimetric analysis, as discussed in the thermal stability section. Following melt mixing, disk-shaped samples with a thickness of 2 mm and a diameter of 25 mm were fabricated using an extrusion–injection process (Micro 5 cc injection molder, DSM) operated at 5 MPa. The temperatures of the injection zone and mold holder were set at 170 °C and 60 °C, respectively, enabling the formation of well-defined specimens for subsequent characterization. Treated fibers (TSCB) were added to the PLA matrix to prepare bio-composites with different compositions (0, 5, 10, and 15 wt%). Table 2, below, summarizes the abbreviations of the formulations prepared via the melt blending route.

#### 2.4.2. Solvent Casting

In addition to the melt processing technique, the materials were also blended using a solvent casting method. Films with fiber contents of 0, 5, 10, and 15 wt% were produced, each with a dry thickness of approximately 2 mm. Chloroform (${CHCL}_{3}$) was used as the solvent, following established procedures [18,19]. To prepare the films, 20 g of PLA was dissolved in 120 mL of chloroform while stirring at 40 °C for 2 h until fully dissolved. After the complete dissolution of PLA, cellulose fibers were added at varying percentages (0, 5, 10, and 15 wt%). To ensure proper dispersion of the fibers within the PLA and to prevent any agglomeration, a sonicator and an Ultraturax were used. These steps also helped to eliminate any air bubbles within the prepared solutions. The mixture was then cast into Petri dishes, where the solvent was allowed to evaporate at room temperature. Once the films were formed, a punch was used to obtain disks with a diameter of approximately 25 mm and a thickness of about 2 mm, as well as tensile specimens with similar dimensions to those obtained via melt processing. This was necessary for a comparative study between the samples produced by the two methods (melt processing and solvent casting). In the following, the terms S-PLA and S-PLA-TSCB5,10,15 are used to define the non-reinforced PLA film, and the PLA composites containing fibers treated at different contents, respectively, as shown in Table 3 below.

#### 2.4.3. Three-Dimensional Printing Process

**Figure 2:**
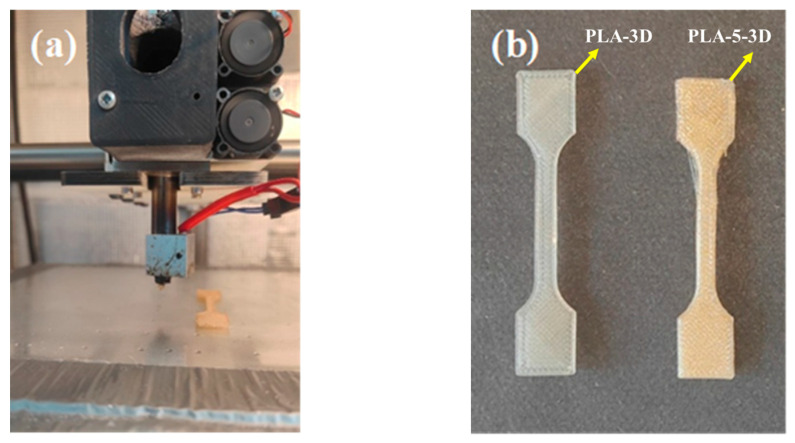
Three-dimensionally printed PLA-based composites: (**a**) printing process; (**b**) printed tensile specimens.

The MEX 3D printing process was performed using a Tobeca system, an extrusion-based additive manufacturing printer equipped with a hopper-fed extruder as illustrated in Figure 2, below. The printer has a build volume of 900 × 400 × 400 mm and was directly fed with the composite pellets previously prepared by melt extrusion. Tensile test specimens were designed using CATIA V5 and exported in STL format. Printing parameters were defined and sliced using Repetier-Host software. The corresponding G-code was then generated and uploaded to the printer to fabricate the test samples. The printing temperature was set to approximately 200 °C, with a build plate temperature of 60 °C, a printing speed of about 35 mm/s, and an infill density of 100%.

### 2.5. Characterization Methods

#### 2.5.1. Differential Scanning Calorimetry (DSC)

The thermal properties of neat PLA and the bio-composites were investigated with the help of a differential scanning calorimeter DSC-3 from Mettler Toledo, equipped with a liquid nitrogen cooling system. The DSC cell was constantly purged with nitrogen at a flow rate of 50 mL/min. A set of heating/cooling ramps was carried out following a three-step process; the samples were first heated to 200 °C and kept in the molten state for 2 min to erase the thermal history of the material. They were then cooled to 25 °C at 10 °C/min to evaluate the ability of the PLA component to crystallize upon cooling. After the cooling treatment, the samples were heated back up to 200 °C at 10 °C/min. The percentage of crystalline was calculated upon the second heating by using Equation (1):

(1) $$X_{c}\left(\%\right)=\frac{{∆H}_{m}-{∆H}_{cc}}{\left(1-w\right)*{∆H{}^{\circ}}_{m}}*100$$

where ${∆H}_{m}$ and ${∆H}_{cc}$ are the melting and the cold crystallization enthalpies (J/g), (w) is the weight fraction of fibers inside the polymer matrix, ${∆H{}^{\circ}}_{m}$ represents the theoretical melting enthalpy for a 100% crystalline PLA (93.6 J.g^−1^) [20].

#### 2.5.2. Scanning Electron Microscopy (SEM)

To investigate the morphology of the PLA/TSCB composite obtained by either melt extrusion or solvent casting, SEM analysis was performed using scanning electron microscopy (JEOL 7600 F FEG) on cryo-fractured samples with an 8-kV accelerating voltage.

Herein, the specimens were cryofractured after immersion in liquid nitrogen for 2 min, and the resulting fracture surfaces were coated with a 15 nm thick gold layer to enhance conductivity prior to observation.

#### 2.5.3. Melt Shear Rheological Measurements

Dynamic oscillatory measurements in the melt state were conducted to evaluate the viscoelastic behavior of the neat polymer and the prepared bio-composites. Prior to testing, the samples were vacuum-dried at 70 °C for 8 h. Rheological analyses were carried out under a nitrogen atmosphere using a Rheometrics ARES rheometer equipped with 25 mm parallel plate geometry.

All the specimens were evaluated at three different temperatures—170 °C, 180 °C, and 190 °C—to evaluate the effect of temperature on the viscoelastic properties of the studied materials, depending on the preparation methods. All specimens were placed between the preheated plates of the rheometer, with the gap fixed at 1.5 mm. A 3 min holding period at the selected temperature was applied to eliminate any residual crystallinity prior to testing, after which the temperature was kept constant throughout the experiment. For each sample, frequency sweep tests ranging from 100 to 0.01 rad/s were carried out within the linear viscoelastic region (5% strain). To ensure reproducibility, all measurements were performed in triplicate.

#### 2.5.4. Mechanical Properties

Mechanical tensile tests were performed in accordance with the ASTM D638 standard using an Instron universal testing machine at room temperature (23 °C) and a crosshead speed of 5 mm/min. The specimens had dimensions of 20 × 4 × 2 mm (length × width × thickness). Prior to testing, the samples were dried in a vacuum oven at 70 °C for 6 h and stored in sealed desiccators to prevent moisture absorption. The reported data represent the average values obtained from five specimens for each sample.

#### 2.5.5. Size Exclusion Chromatography (SEC)

The weight-average molecular weight ($\bar{M}w$), number average molecular weight ($\bar{M}n$) and dispersity (D) of neat PLA, as well as all the PLA extruded from the prepared bio-composites, and after processing at different temperatures, were measured by SEC on a system equipped with a Shimadzu LC 20AD pump and a Perkin Elmer Series 200 automatic injector. The separation was performed using a pre-column (PL gel 5 μm Guard, 50 × 7.5 mm) followed by three columns (PL gel 5 μm Mixed C, 300 × 7.5 mm, polystyrene/divinylbenzene). The system included a Shimadzu CTO 20A oven set to 30 °C. Detection was carried out with a Shimadzu SPD 20A UV detector (λ = 280 nm), a Wyatt TREOS light scattering detector (3 angles), a Wyatt ViscoStar II viscometer, and a Shimadzu RID 10A refractometer. Chloroform was used as the eluent. Samples were prepared at a concentration of 1 mg.mL^−1^ and filtered through a 0.45 μm pore-size membrane before injection. Data acquisition and analysis were conducted using the ASTRA 6 software.

#### 2.5.6. Density Measurements

Based on each reinforcement component’s weight percentage and density, theoretical density ($\rho_{thy}$) (g/cm^3^) of the composites was determined firstly using Equation (2):

(2) $$\frac{1}{\rho_{thy}}=\sum \frac{W_{i}}{\rho_{i}}.$$

where $W_{i}$ is the weight fraction of the polymer matrix and sugarcane *bagasse* (TSCB), while $\rho_{i}$ is the density of the PLA polymer matrix and the fibers. $\rho_{PLA}$ and $\rho_{TSCB}$ are 1.24 and 1.25 g/cm^3^, respectively.

Secondly, the experimental density (g/cm^3^) was calculated with respect to ASTM D1895. The density was determined using Equation (3) with the average values from three different samples.

(3) $$\rho_{exp}=\frac{m}{V}$$

The mass (m) of both neat PLA and composite samples was determined using a precision balance, whereas their volume (v) was calculated based on dimensional measurements taken with a digital pycnometer.

Then, the void content of neat PLA and the three composite formulations was calculated based on theoretical and experimental density values, following the ASTM D2734–94 standard, as presented in Equation (4).

(4) $$\mathrm{Porosity}=(1-\frac{\rho_{exp}}{\rho_{thy}})\times 100$$

## 3. Results and Discussion

### 3.1. Physical Properties of the TSCB Fibers

#### Granulometric Distribution

**Figure 3:**
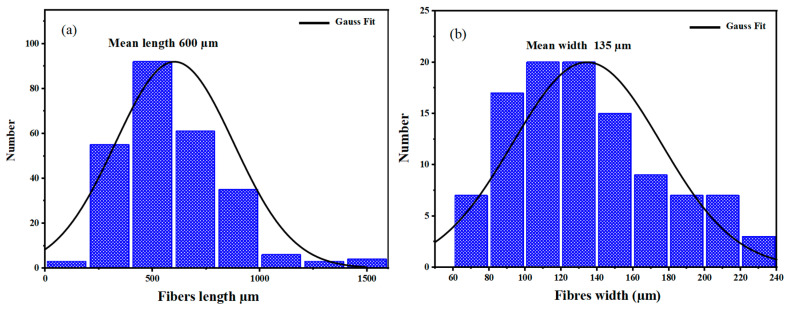
Size distribution histograms of the (**a**) length and (**b**) width of the treated sugarcane *bagasse* fibers (TSCB).

The fiber size distribution of the sugarcane *bagasse* (TSCB) is shown in Figure 3. This analysis aimed to determine the average length and diameter of the extracted fibers by using optical microscopy coupled with image analysis. A total of 200 individual fibers were measured, and the resulting data were processed using the ImageJ software (version 1.54p) to obtain their size distribution profiles. This step was crucial for estimating the maximum fiber content that could be incorporated into the PLA matrix without compromising the material’s integrity. In particular, it helped anticipate potential issues such as fiber agglomeration or the formation of aggregates within the final composite. This upper limit is defined by a theoretical percolation threshold, which can be estimated using the following Equation (5), below [21,22,23,24]:

(5) $$\Psi \;=\;\frac{0.7}{\frac{L}{D}}$$

In this context, ψ represents the theoretical volume fraction corresponding to the percolating filler network, while P = L/D denotes the aspect ratio of the TSCB fibers, where L is the average length (Figure 3a) and D the average diameter (Figure 3b). Based on these measurements ($\frac{L}{D}=4.45)$, the theoretical percolation threshold was estimated at approximately 15 wt%. Taking this threshold into account, three fiber contents were selected for incorporation into the PLA matrix: 5%, 10%, and 15%. These values were chosen to assess how varying fiber content influences the properties of the resulting composites. The fibers were introduced into the PLA using two previously described methods: solvent casting and melt processing. This allowed for a direct comparison of both techniques in terms of their efficiency in dispersing the fibers and ensuring uniform integration within the PLA matrix.

### 3.2. Thermal Stability and Rheology of the Bio-Composites Based on PLA Composites

#### 3.2.1. Thermal Stability of PLA and Bio-Composites Prepared by Melt Processing and Solvent Routes

**Figure 4:**
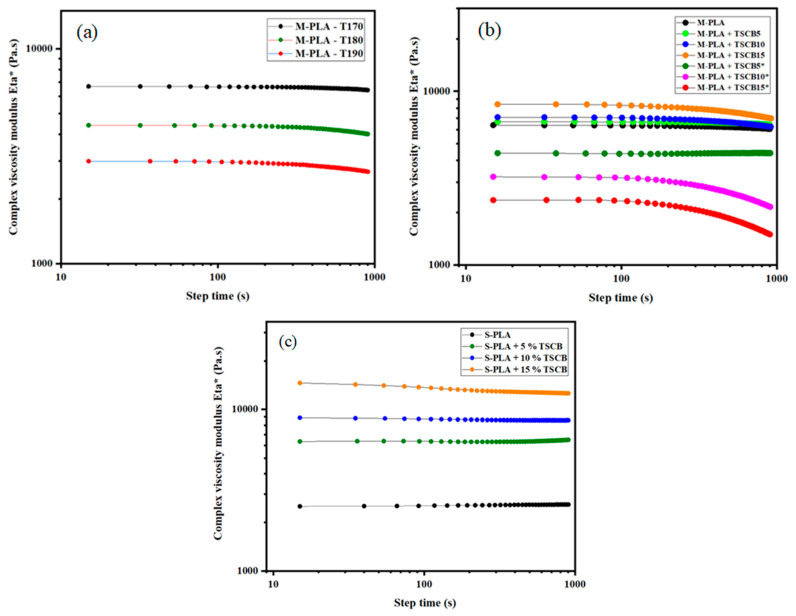
Results of (**a**) complex viscosity modulus versus time t of neat PLA at various temperatures. (**b**) Influence of fiber surface treatment on the viscosity modulus versus time of PLA-TSCB bio-composites prepared in the molten state at 170 °C (the * indicates the non-neutralized fibers). (**c**) Evolution of complex viscosity of PLA and its bio-composites prepared by solvent casting at 170 °C.

Figure 4a shows how the complex viscosity modulus of neat PLA changes over time at three processing temperatures: 170 °C, 180 °C, and 190 °C. At all temperatures, PLA remained thermally stable during the first 5 min of testing, with no noticeable loss in mass or viscosity. This confirms that PLA can tolerate short processing times without significant thermal degradation. After this period, a slight decrease in viscosity was observed, especially at 190 °C. These results demonstrate that PLA shows good thermal stability under the selected processing conditions, particularly during the early stages of processing, which align with previous findings reported in the literature [6,7]. Based on these results, we selected the most favorable experimental conditions for twin-screw extrusion, with a processing temperature of around 170 °C and a screw speed of 100 rpm for 3 min. These conditions would help prevent PLA degradation and preserve its properties during processing. The rheological results thus highlight the importance of balancing efficient processing with the thermal stability of sensitive biodegradable polymers such as PLA [3,25].

**Figure 5:**
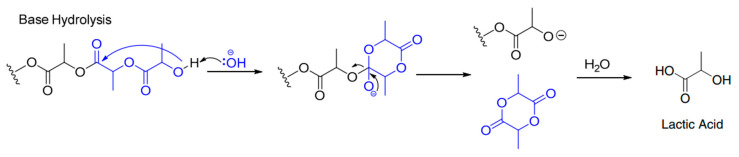
Chemical reaction mechanism of PLA degradation in the presence of a strong base (NaOH) [26].

Polylactic acid (PLA) is known for biodegradability but suffers from limited thermal stability and sensitivity to hydrolytic degradation; exposure to strong alkaline environments can greatly accelerate its breakdown. In this study, sodium hydroxide was used to treat *bagasse* fibers, yet the neutralization step left residual alkalinity on the fiber surfaces; once incorporated into the PLA matrix, these untreated zones created localized high-pH regions and initiated PLA depolymerization through the alkaline degradation mechanism illustrated in Figure 5 as explained by McKeown and al [26], undermining composite performance and durability.

In the molten state, the rheological behavior of PLA composites is strongly influenced by the surface condition of the fibers. As shown in Figure 4b, composites containing non-neutralized fibers (TSCB5*, TSCB10*, and TSCB15*) exhibit a pronounced decrease in viscosity, which can be attributed to hydrolytic degradation caused by residual NaOH from the alkaline treatment. Conversely, thorough neutralization of the fibers results in significantly higher viscosity values in the corresponding composites (TSCB5, TSCB10, and TSCB15), reflecting reduced degradation and improved fiber–matrix interactions. These results underline the critical role of proper fiber surface treatment in maintaining thermal and rheological stability during melt processing. When considering the solvent-casting route and after neutralizing the fiber surface to overcome any other degradation of PLA, a markedly different behavior is observed. The complex viscosity of S-PLA and its solvent-cast bio-composites increases noticeably with fiber addition, owing to the structural reinforcement and improved dispersion provided by the fibers. As illustrated in Figure 4c, solvent-cast composites display substantially higher initial viscosity than their melt-extruded counterparts (e.g., the S-PLA composite with 15% of fibers reaches about 14 500 Pa.s at t = 0, compared to roughly 8 400 Pa.s for the same formulation processed by melt state). This difference is mainly due to the absence of thermomechanical stresses during solvent casting, which preserves fiber integrity and enhances fiber–matrix interactions. Over 15 min at 170 °C, the viscosity remains stable for all formulations except the one containing 15% of TSCB, where a noticeable drop is observed, attributed to hydrolytic degradation initiated by the cut of the ester link and the interaction of fiber hydroxyl groups and the carboxyl end groups of PLA [14,27]. The pronounced decrease in viscosity observed for composites containing non-neutralized fibers can also be explained by the presence of unbound or weakly bonded fibers within the PLA matrix. This can be attributed to insufficient interfacial wetting caused by residual alkalinity, which limits fiber–matrix adhesion and promotes local degradation, thereby reducing the rheological stability of the melt.

These four indicate that after twin-screw extrusion at 170 °C, PLA showed a slight decrease in molecular weight, on the order of 10%. However, after twin-screw extrusion (E) and rheological measurements (R), PLA presented a 20% reduction in its molecular weight ($\bar{M}n,\;\bar{M}w\;\mathrm{and}\;\bar{M}z)$ as mentioned in Table 4 below.

To thoroughly examine the effect of temperature and processing factors, we evaluate a parameter “K” (Equation (6)) as the degradation factor [28]:

(6) $$K\;=\;\frac{\bar{M}n\;(pellets)}{\bar{M}n\;(Processed-polymer)}$$

where $\bar{M}n\;(pellets)$ represents the average molecular weight of the neat polymer and $\bar{M}n\;(Processed-polymer)$ is the average molecular weight of the processed polymer at temperature T. The results showed that “K” values for PLA increased slightly with rising processing temperatures, indicating a reduction in average molecular weight. Specifically, PLA exhibited noticeable thermal degradation at 170 °C and 190 °C after 3 min of processing, as shown in Figure 4a.

This section clearly demonstrates the influence of the chemical treatment on the stability of the composite under thermomechanical stresses. Therefore, only the treated and neutralized fiber surface (TSCB) will be considered for the rest of the study.

#### 3.2.2. Small-Amplitude Oscillatory Shear (SAOS) Rheology of Molten and Solvent Casting States

**Figure 6:**
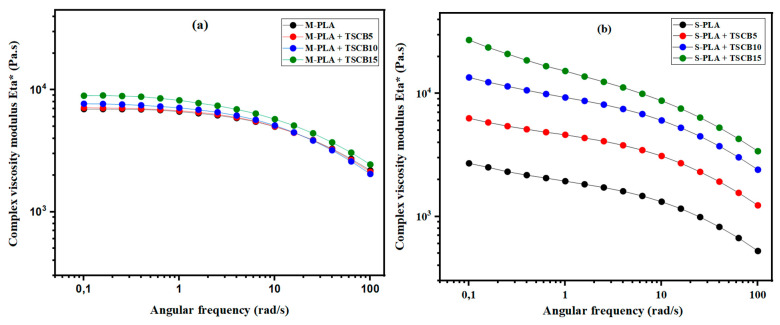
Complex viscosity modulus (Eta*) versus angular frequencies of PLA and its bio-composites prepared by (**a**) molten state and (**b**) solvent route at 170 °C.

Figure 6a presents the evolution of the complex viscosity (η*) as a function of frequency for neat PLA and its bio-composites reinforced with sugarcane *bagasse* fibers (TSCB) prepared via the melt-processing route. All samples exhibit a nearly flat plateau in the low- to mid-frequency range, followed by a clear shear-thinning behavior at higher frequencies. The incorporation of TSCB fibers systematically increases viscosity across the entire frequency spectrum, with the effect becoming more pronounced as fiber content rises. This behavior is characteristic of reinforced composites, where strong fiber–matrix interactions hinder polymer chain mobility, leading to higher flow resistance. As fiber loading increases, the non-Newtonian character of the composites becomes more evident, especially in the high-frequency region where the applied shear partially disrupts the internal fiber–matrix network.

In contrast, Figure 6b reports the rheological behavior of PLA and its bio-composites produced via the solvent-casting route at 170 °C. The curves also display a shear-thinning profile, but the underlying mechanisms differ slightly. At low frequencies, the increase in viscosity with fiber content is more pronounced, primarily due to dominant filler–filler interactions that form an interconnected network within the polymer matrix. This behavior can also be explained by the good dispersion of fibers within the PLA matrix achieved through solvent casting, which promotes more effective stress transfer and enhances the continuity of the internal network. As the fiber concentration increases, the interparticle distance decreases, resulting in more frequent fiber–fiber collisions and amplifying the rheological response [29]. This effect is further strengthened by the disruption of PLA chain orientation, which limits molecular mobility. At higher frequencies, hydrodynamic interactions begin to play a greater role, although their influence is partially masked by the persistence of fiber–fiber contacts.

When considered together, Figure 6a,b highlight that, regardless of processing route, adding rigid TSCB fibers substantially increases the complex viscosity of PLA and modifies its viscoelastic profile. However, the solvent-cast composites tend to show a more pronounced viscosity rise at low frequencies, reflecting stronger filler–filler network effects, whereas melt-processed composites display a more balanced contribution between fiber–matrix interactions and hydrodynamic effects. These distinctions underline the impact of processing method on the microstructure and rheological response of the composites, with direct implications for their processability.

### 3.3. Morphological Analysis of Bio-Composites Produced by Melt Processing and Solvent Route

**Figure 7:**
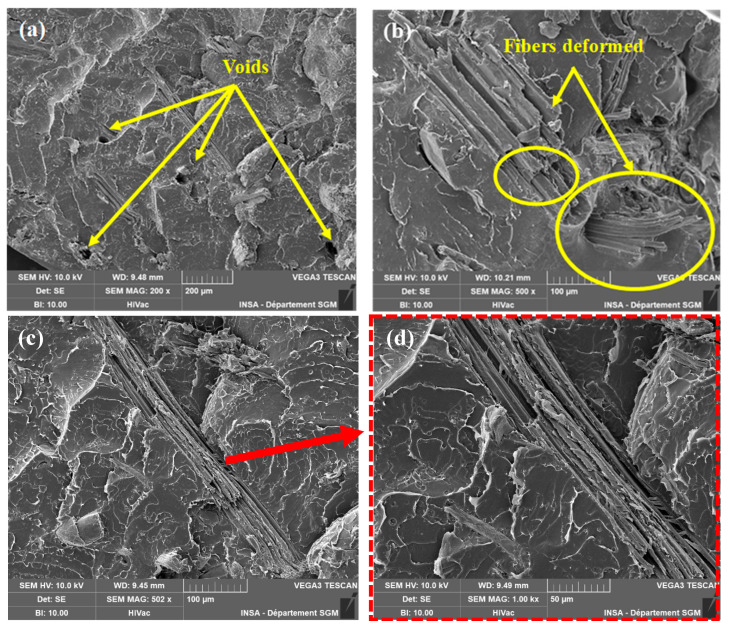
SEM micrographs of the fracture surfaces of PLA + 15 wt.% TSCB composites processed via melt blending (**a**,**b**) and solvent casting (**c**,**d** (close-up view)).

To better understand the internal morphology of PLA/TSCB composites, both melt-processed and solvent-cast samples were examined by SEM after cryo-fracturing in liquid nitrogen and applying a thin gold coating to enhance surface conductivity. For the melt-processed samples, the fibers appeared fairly well dispersed within the PLA matrix; however, several pull-out marks and interfacial voids were observed (Figure 7a), suggesting incomplete fiber embedding and moderate interfacial adhesion. In addition, the high shear forces involved in extrusion contributed to a reduction in fiber size and partial deformation of their structure, as illustrated in Figure 7b. In contrast, the solvent casting technique yielded a more uniform morphology, with fibers homogeneously distributed throughout the matrix and retaining their original dimensions (Figure 7c). This method, which avoids mechanical and thermal stresses, enabled better control over fiber dispersion and effectively preserved fiber integrity (Figure 7d). Overall, solvent casting provided superior uniformity and fiber preservation, while melt processing caused some structural alteration but still achieved an acceptable dispersion quality.

### 3.4. Differential Scanning Calorimetry Properties of the Bio-Composites

To study the effect of the addition of *bagasse* fibers on the thermal properties of PLA, a series of Differential Scanning Calorimetry (DSC) analyses was carried out, the protocol for which was described in Section 2. The results obtained from this analysis are reported in terms of glass transition temperature $\left(T_{g}\right)$, crystallization temperature $\left(T_{c}\right)$, melting temperature $\left(T_{m}\right)$, fusion enthalpy $\left({\Delta H}_{m}\right)$, cold crystallization enthalpy ${\Delta H}_{cc}$, and crystallinity index ($X_{c}$).

#### Melt Processing and Solvent Casting Properties

**Figure 8:**
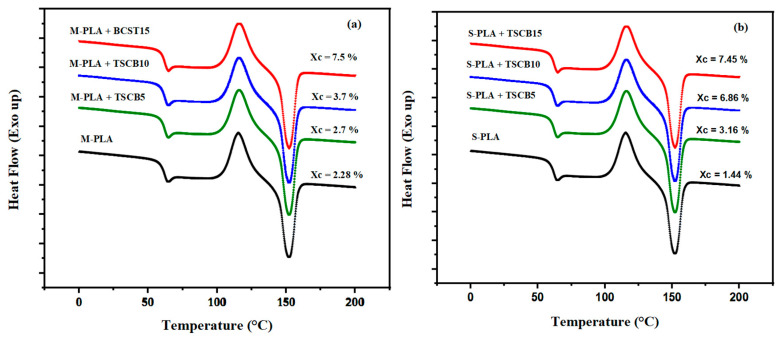
DSC thermograms (second heating cycle) of neat PLA and PLA/TSCB bio-composites prepared via (**a**) melt processing and (**b**) solvent casting method.

Figure 8 presents the DSC thermograms (second heating cycle) of neat PLA and PLA/TSCB bio-composites prepared via both melt processing and solvent casting. The corresponding thermal parameters are summarized in Table 5 and Table 6. In the melt-processed samples (Figure 8b), a distinct crystallization peak was observed for all compositions. For neat PLA (M-PLA), the crystallization temperature (Tc) appeared around 119 °C. With the incorporation of treated sugarcane *bagasse* (TSCB) fibers, Tc exhibited a slight increase, indicating that the dispersed fibers acted as nucleating agents, promoting earlier crystallization. This effect intensified with higher fiber loadings, as reflected by the rise in the crystallinity index (Xc), which increased from 2.28% for neat PLA to 7.5% for PLA/15% TSCB. The glass transition temperature (Tg), however, remained stable around 60 °C for all melt-processed formulations, suggesting that fiber addition did not influence the mobility of PLA chains in this configuration.

In contrast, the solvent casting method produced slightly different thermal behavior, as illustrated in Figure 8a, where a moderate decrease in Tg was observed for neat PLA (F-PLA), approximately 57 °C, likely due to residual solvent acting as a plasticizer. With increasing fiber content, Tg gradually rose back toward 60 °C. Notably, the crystallinity index of the S-PLA-TSCB10 sample (10% TSCB) was surprisingly higher than that of its melt-processed counterpart (M-PLA-TSCB10), even though this composition is close to the percolation threshold. Despite the absence of significant changes in Tc for S-PLA-TSCB10, its higher Xc value suggests that the fibers remained more intact and better distributed, effectively enhancing nucleation. Furthermore, the cold crystallization enthalpy (ΔHcc) values obtained for the solvent-cast composites were consistently lower compared to those processed by melt blending, reflecting a more advanced crystalline structure likely promoted by the solvent route. Overall, both methods confirm the nucleating effect of TSCB fibers, but solvent casting appears to promote better crystallization under specific conditions, especially at intermediate fiber loadings (close to percolation).

### 3.5. Mechanical Properties

#### Tensile Test Properties

**Figure 9:**
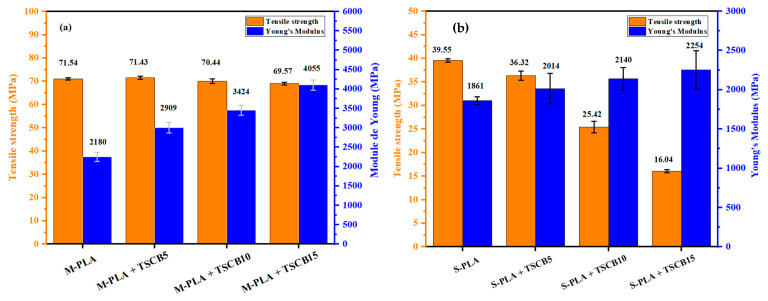
Evolution of Young’s modulus and tensile strength of neat PLA and PLA/TSCB bio-composites prepared via (**a**) melt processing and (**b**) solvent casting method.

The mechanical properties of pure PLA and the PLA/TSCB bio-composites prepared by either solvent casting or melt extrusion are summarized in Figure 9, as well as in Table 7. Although PLA is a brittle material, it exhibits a high modulus and strong tensile strength [30]. The M-PLA/TSCB (Figure 9a) composites showed an increase in Young’s modulus compared to pure PLA due to the rigidity of the cellulose fibers, indicating an enhanced stiffness of the final material. For the specimens prepared by melt processing, neat M-PLA exhibited a Young’s modulus of approximately 2180 MPa, while PLA reinforced with 15% fibers reached 4055 MPa, indicating an improvement of about 86%. In contrast, for the PLA specimens prepared by solvent casting, as shown in Figure 9b, the Young’s modulus was around 1861 MPa, whereas the PLA + 15% fiber composite achieved approximately 2254 MPa, corresponding to an increase of about 21%

However, the elongation at break decreased with the addition of TSCB to the PLA matrix. This reduction can be attributed to the high stress concentration around the filler particles, leading to delamination between PLA and TSCB and subsequent material fracture. The interface between PLA and TSCB did not exhibit adequate adhesion due to the polar hydroxyl groups in the filler, and as a result, stress could not be efficiently transferred across the interface.

It was also clear that the maximum tensile strength decreased as the fiber content in the blends increased. For example, it dropped from 39.55 MPa for neat S-PLA to 16.04 MPa for the S-PLA + 15% TSCB. This reduction can be attributed to the insufficient dispersion of fibers in the matrix, caused by fiber-fiber interactions hindering the efficient transfer of stress from the matrix to the fibers. This effect became even more pronounced as the fiber content in the material increased. However, it is interesting to note that for samples prepared via melt processing, the tensile strength showed a smaller decrease with increasing fiber content, indicating better dispersion and integration of the fibers into the matrix. In all the bio-composites, the tensile strength and elongation at break slightly decreased, but Young’s modulus went up to a small degree compared with pure PLA, thus corroborating the research by Abir and al [13]. The increase in Young’s modulus indicated an enhanced stiffness in the materials, which can be particularly beneficial for applications where material rigidity and hardness are essential. Thus, despite the slight reduction in tensile strength, these bio-composites offer a significant advantage in fields requiring stiffer and harder materials, optimizing their use in specific industrial applications.

### 3.6. Comparative Study Between Mechanical Properties of Printed and Injected Specimens

In order to better understand the influence of processing technique on the final properties of the developed composites, a comparative study was conducted between injection-molded (M-PLA) and 3D-printed specimens (M-PLA-3D) at different fiber content (5, 10, and 15 wt%).

#### 3.6.1. Density and Void Content

Table 8 presents theoretical and experimentally measured densities, along with the corresponding porosity values calculated using Equation (4), for the injected and 3D-printed PLA-based bio-composites. As expected, the overall density of the composites increases with the addition of sugarcane *bagasse* fibers due to the higher intrinsic density of the lignocellulosic filler. However, a clear discrepancy is observed between the theoretical and measured densities, particularly in the 3D-printed samples. This difference indicates the presence of internal voids, which are mainly attributed to the printing process itself. During 3D printing, the layer-by-layer deposition often leads to incomplete fusion between adjacent lines and the formation of inter-bead gaps. These structural irregularities contribute significantly to the overall porosity of the printed materials. In contrast, injection-molded samples show better compaction and reduced porosity. Similar void structures have been reported in 3D-printed PLA reinforced with glass fibers [31], supporting the conclusion that additive manufacturing can result in materials with higher internal porosity that negatively affects their mechanical integrity.

#### 3.6.2. Mechanical and Morphological Properties

**Figure 10:**
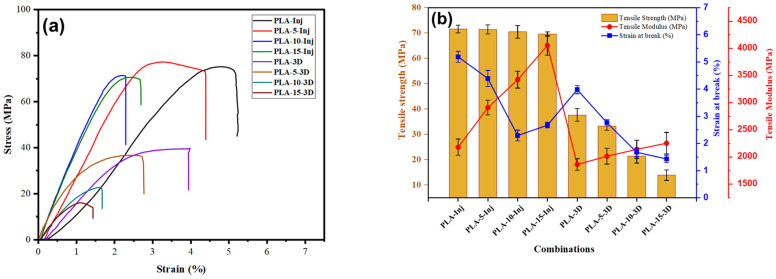
Representation of (**a**) stress–strain curves of injection-molded and 3D-printed specimens and (**b**) tensile strength, Young’s modulus, and elongation at break of injection-molded and 3D-printed specimens.

Figure 10a presents the tensile stress–strain curves of M-PLA composites produced by injection molding and 3D printing. The majority of the samples showed a typical mechanical response characterized by an initial linear elastic region, followed by plastic deformation up to the maximum stress point. Beyond this peak, the stress gradually decreased with increasing strain until final failure occurred.

Figure 10b shows the tensile Young’s modulus, tensile strength, and strain at break of the printed and injected PLA composites obtained from the stress–strain curves. It is clear that the Young’s modulus of the printed M-PLA-3D is 1.86 GPa, which is much lower than the value found by injection molding (2.18 GPa). This reduction in mechanical properties could be explained by the void formation between printed layers and beads, increased porosity in the material produced by the printing process [32]. In addition, changes in the degree of crystallinity during the 3D printing process could also have affected the material’s mechanical performance [33]. It can also be seen from Figure 10b that the Young’s modulus of the 3D-printed composites is lower than that of the injection molded composites. This observation is explained by the fact that increasing fiber content could lead to voids generation, mitigating the final stiffness of the 3D-printed materials regarding the injected molded samples. Additionally, it can be seen that the maximum tensile strength of the printed M-PLA-3D (37.66 MPa) is slightly lower than that found by injection molding (71.54 MPa), which is attributed to the voids between layers. However, for M-PLA injected specimens, they present a higher tensile strength than that of the printed M-PLA-3D samples. Moreover, the strain at break of the M-PLA-15TSCB is 2.67% as shown in Figure 10b below, which is 86.7% higher than that of the printed pristine M-PLA-3D (1.43%). This improvement in strength and ductility is attributed to the sugarcane *bagasse* fibers acting as reinforcement and delaying crack growth. Moreover, the strain at break decreases with the increase in fiber content. This observation is attributed to fiber agglomeration and increased porosity.

**Figure 11:**
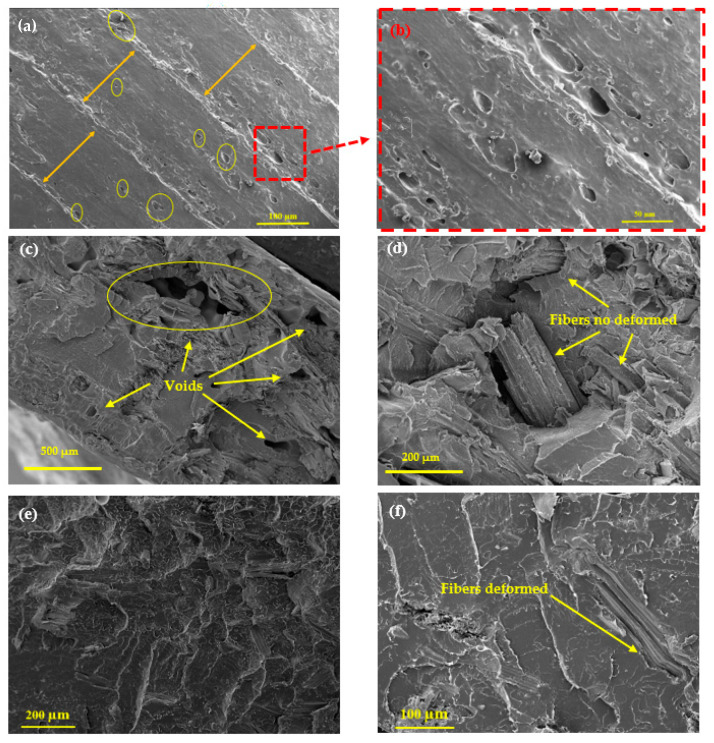
SEM Images of the fracture surface after tensile testing of the 3D-printed samples (**a**) PLA-5-3D; (**b**) PLA-5-3D (close-up view); (**c**) PLA-15-3D; (**d**) PLA-15-3D (close-up view); and injected specimens (**e**) PLA-15-inj and (**f**) PLA-5-inj.

Figure 11 presents SEM images of the fracture surfaces of 3D-printed and injected PLA composites after tensile testing. As shown in Figure 11a, a visible separation occurs between adjacent printed beads, which is mainly attributed to poor inter-layer bonding during the 3D printing process and to stress concentrations around voids formed during fabrication [34,35]. This kind of debonding has already been reported in printed PLA systems [36]. Such defects can be caused by the presence of voids and shrinkage of the extruded filaments, which are linked to the semicrystalline nature of PLA [37]. These phenomena reduce the contact surface between adjacent beads, weakening the interface and making it prone to separation when subjected to mechanical stress. Moreover, Figure 11c reveals the presence of internal voids within the deposited beads, which are mainly linked to the presence of large fillers and to local fiber agglomeration. Although such pores and interlayer defects are inherent to the 3D-printing process, the material extrusion route nevertheless preserves the original morphology of the *bagasse* fibers, as clearly observed in Figure 11d. In contrast, the micrographs of the injected specimens reveal the absence of pores or voids (Figure 11e), which explains the good mechanical performance obtained through this processing route. Despite this densification, the fibers exhibit some deformation in their structure due to the shear and thermomechanical stresses applied during injection molding, as illustrated in Figure 11f. This distinction highlights the ability of 3D printing to maintain the intrinsic structural integrity of the natural fibers as mentioned in Figure 11d, despite the presence of printing-related defects. Additionally, future work could benefit from multiscale numerical modeling to better understand stress distribution and damage evolution within PLA/TSCB composites, following approaches similar to those used for continuous-fiber reinforced structures and advanced composite architectures [38,39]. 

## 4. Conclusions

In conclusion, this study investigated the dispersion behavior of alkali-treated sugarcane *bagasse* fibers (TSCB) in a bio-based and biodegradable PLA matrix by comparing two processing routes: melt blending and solvent casting. The solvent-casting approach, considered here as a model method, enabled the preservation of fiber integrity due to the absence of thermomechanical stresses. However, its reliance on organic solvents raises environmental and health concerns and limits its feasibility for large-scale or industrial production. Conversely, the melt-blending route proved to be a more realistic and application-oriented process, providing enhanced fiber dispersion, improved densification, and superior mechanical properties despite the possibility of moderate fiber damage. Overall, the melt-processed composites exhibited more reliable structural homogeneity and performance, reinforcing their suitability for practical applications where scalable and environmentally acceptable fabrication methods are required. As a further step, the study evaluated the feasibility of using these composites in 3D printing. Although the 3D-printed specimens exhibited lower mechanical performance than their injection-molded counterparts due to higher porosity and weaker interlayer cohesion, the results highlight important advantages associated with the additive manufacturing route. The material extrusion process preserves the intrinsic morphology of the *bagasse* fibers, which is not the case for injection molding, where strong thermo-mechanical stresses tend to deform and damage the fibrillar structure. This preservation of the natural fibers, combined with the ability of 3D printing to fabricate complex geometries with reduced material waste, reinforces its relevance for the development of sustainable bio-composites. Despite the presence of printing-related defects, 3D printing therefore remains a promising and versatile technique, fully aligned with the objectives of environmental sustainability and the advancement of circular bio-based materials.

## Figures and Tables

**Table 1:** Main characteristics of PLA.

Polymer 10.	Supplier	Tg (°C)	Tm (°C)	MFI (g/10 min) (190 °C/2.16 Kg)	Density (g/cm^3^)
PLA	Natureworks, Plymouth, MN, USA	60	155	6	1.24

**Table 2:** Composition of PLA-based composite reinforced with sugarcane *bagasse* fibers (TSCB) prepared by melt processing.

Sample Name	PLA Content (wt%)	TSCB Content (wt%)
M-PLA	100	0
M-PLA-TSCB5	95	5
M-PLA-TSCB10	90	10
M-PLA-TSCB15	85	15

**Table 3:** Composition of PLA-based composite reinforced with sugarcane *bagasse* fibers (TSCB) prepared by solvent casting.

Sample Name	PLA Content (wt%)	TSCB Content (wt%)
S-PLA	100	0
S-PLA-TSCB5	95	5
S-PLA-TSCB10	90	10
S-PLA-TSCB15	85	15

**Table 4:** Characteristic of average molecular weights obtained by SEC of different samples at various temperatures up to extrusion (E) and rheology measurements (R).

Samples	$\bar{M}w$ (g/mol)	$\bar{M}n$ (g/mol)	$\bar{M}z$ (g/mol)	K (Degradation Parameter)
PLA pellet	125.000	91.540	168.100	
PLA E@ 170 °C	122.400	90.550	160.700	1.01
PLA R@ 190 °C	117.200	87.760	152.900	1.04
PLA5 E@ 170 °C	111.900	88.320	146.800	1.03
PLA5 R@ 190 °C	106.600	82.650	141.000	1.10
PLA10 E@ 170 °C	108.200	82.400	144.300	1.11
PLA10 R@ 190 °C	97.930	79.100	138.300	1.16
PLA15 E@ 170 °C	107.200	80.590	142.800	1.13
PLA15 R@ 190 °C	98.670	77.630	125.600	1.17

**Table 5:** Thermal characteristics of M-PLA/TSCB blends prepared by melt processing with different compositions.

Sample	$T_{g}$ (°C)	$T_{c}$ (°C)	$T_{m}$ (°C)	${\Delta H}_{m}PLA$ $(J.g^{-1}$)	${\Delta H}_{cc}PLA$ $(J.g^{-1}$)	$X_{c}$ (%)
M-PLA	60	119	150	20.57	18.43	2.28
M-PLA + TSCB5	60	120	150.9	21.5	19.09	2.7
M-PLA + TSCB10	60.3	121	150.9	20.61	17.47	3.7
M-PLA + TSCB15	60.1	121	151.1	22.77	18.44	7.5

**Table 6:** Thermal characteristics of S-PLA/TSCB blends prepared by solvent casting with different compositions.

Sample	$T_{g}$ (°C)	$T_{c}$ (°C)	$T_{m}$ (°C)	${\Delta H}_{m}PLA$ $(J.g^{-1}$)	${\Delta H}_{cc}PLA$ $(J.g^{-1}$)	$X_{c}$ (%)
M-PLA	57	119	151.9	9.73	8.38	1.44
S-PLA + TSCB5	58	121	151.8	15.22	12.41	3.16
S-PLA + TSCB10	59.8	121	150.9	17.82	12.04	6.86
S-PLA + TSCB15	60.1	122	150.9	18.04	12.11	7.45

**Table 7:** Strain at yield (%) for PLA and all bio-composites.

Method	Sample	Strain at Yield (%)
Melt processing	M-PLA	5.19 (±1.2)
	M-PLA + TSCB5	4.39 (±0.7)
	M-PLA + TSCB10	2.29 (±0.5)
	M-PLA + TSCB15	2.67 (±0.3)
Solvent casting	S-PLA	3.34 (±0.7)
	S-PLA + TSCB5	3.45 (±0.4)
	S-PLA + TSCB10	2.39 (±0.2)
	S-PLA + TSCB15	2.85 (±0.4)

**Table 8:** Density and porosity of the injected and 3D-printed samples.

Formulations	Theoretical Density (g/cm^3^)	Experimental Density (g/cm^3^)	Void Content (%)
M-PLA inj	1.24	1.22	1.6
M-PLA-3D	1.24	1.16	6.5
M-PLA+5 TSCB inj	1.25	1.21	3.3
M-PLA+5 TSCB-3D	1.25	1.15	8.1
M-PLA+10 TSCB	1.26	1.21	4.1
M-PLA+10 TSCB-3D	1.26	1.14	9.7
M-PLA+15 TSCB	1.27	1.18	7.3
M-PLA+15 TSCB-3D	1.27	1.12	12.1

## Data Availability

The original contributions presented in this study are included in the article. Further inquiries can be directed to the corresponding author.

## Supplementary Materials

The following supporting information can be downloaded at: https://www.mdpi.com/article/10.3390/polym18010128/s1, Figure S1: Image of fiber distribution by optical microscope, Figure S2: FTIR spectra of untreated and treated bagasse fibers Figure S3: Typical TGA and DTG results for bagasse fibers (SCB), Figure S4: SEM images of (a) untreated, (b) after alkali treatment SCB fibers and (c) and (d) present SEM images showing the cross-section of an individual SCB fiber; Table S1: Identification of peak positions and corresponding chemical groups in both untreated and treated SCB fibers.

## References

[1] Qiao H., Sudre G., Maazouz A., Lamnawar K. (2025). Dual enhancement of dispersion of cellulose nanocrystals, crystallization performance, flexibility and barrier properties through polyethylene glycol coating in melt-processed poly (L-lactide)-based nanocomposites. Ind. Crops Prod..

[2] Rahmanifard M., Khademi S.M.H., Asheghi-Oskooee R., Farizeh T., Hemmati F. (2024). Reactive processing-microstructure-mechanical performance correlations in biodegradable poly (lactic acid)/expanded graphite nanocomposites. RSC Adv..

[3] Qiao H., Maazouz A., Lamnawar K. (2022). Study of Morphology, Rheology, and Dynamic Properties toward Unveiling the Partial Miscibility in Poly (lactic acid)—Poly (hydroxybutyrate-co-hydroxyvalerate) Blends. Polymers.

[4] Furet A., Singh S., Gardrat C., Alembik L., Jaouannet R., Poças F., Coma V. (2024). Cellulose trays with PLA-based liners as single-used food packaging: Characterization, performance and migration. Food Packag. Shelf Life.

[5] Dejene B.K., Gudayu A.D., Abtew M.A. (2024). Development and optimization of sustainable and functional food packaging using false banana (Enset) fiber and zinc-oxide (ZnO) nanoparticle-reinforced polylactic acid (PLA) biocomposites: A case of Injera preservation. Int. J. Biol. Macromol..

[6] Rajeshkumar G., Seshadri S.A., Devnani G.L., Sanjay M.R., Siengchin S., Maran J.P., Al-Dhabi N.A., Karuppiah P., Mariadhas V.A., Sivarajasekar N. (2021). Environment friendly, renewable and sustainable poly lactic acid (PLA) based natural fiber reinforced composites – A comprehensive review. J. Cleaner Prod..

[7] Al-Itry R., Lamnawar K., Maazouz A. (2014). Reactive extrusion of PLA, PBAT with a multi-functional epoxide: Physico-chemical and rheological properties. Eur. Polym. J..

[8] Jaouadi N., Jaziri M., Maazouz A., Lamnawar K. (2023). Biosourced Multiphase Systems Based on Poly(Lactic Acid) and Polyamide 11 from Blends to Multi-Micro/Nanolayer Polymers Fabricated with Forced-Assembly Multilayer Coextrusion. Int. J. Mol. Sci..

[9] Ait-Abdellah A., Belcadi O., Balla M.A., Bounouader H., Kaddami H., Abidi N., Arrakhiz F.-E. (2024). Alkaline Treatment of Sugarcane Bagasse Fibers for Biocomposite Applications. Cellul. Chem. Technol..

[10] Banerjee A., Jha K., Petru M., Kumar R., Sharma S., Saini M.S., Mohammed K.A., Kumar A., Abbas M., Tag-Eldin E.M. (2023). Fabrication and characterization of weld attributes in hot gas welding of alkali treated hybrid flax fiber and pine cone fibers reinforced poly-lactic acid (PLA) based biodegradable polymer composites: Studies on mechanical and morphological properties. J. Mater. Res. Technol..

[11] Belcadi O., Balla M.A., Legrand C., Lyagoubi N., Laaguel F.E., Khalij L., Desilles N., Gautrelet C., El Minor H., Arrakhiz F.E. (2025). Impact of process parameters and coupling agent on the thermal stability of argan nutshell composites. J. Compos. Mater..

[12] Mazzanti V., Pariante R., Bonanno A., Ruiz de Ballesteros O., Mollica F., Filippone G. (2019). Reinforcing mechanisms of natural fibers in green composites: Role of fibers morphology in a PLA/hemp model system. Compos. Sci. Technol..

[13] Khan A., Sapuan S.M., Zainudin E.S., Zuhri M.Y.M. (2024). Physical, mechanical and thermal properties of novel bamboo/kenaf fiber-reinforced polylactic acid (PLA) hybrid composites. Compos. Commun..

[14] Khemakem M., Lamnawar K., Maazouz A., Jaziri M. (2018). Biocomposites based on polylactic acid and olive solid waste fillers: Effect of two compatibilization approaches on the physicochemical, rheological, and mechanical properties. Polym. Compos..

[15] Phiri R., Rangappa S.M., Siengchin S. (2024). Agro-waste for renewable and sustainable green production: A review. J. Cleaner Prod..

[16] Phiri R., Rangappa S.M., Siengchin S. (2024). Sugarcane bagasse for sustainable development of thermoplastic biocomposites. Ind. Crops Prod..

[17] Taha I., Steuernagel L., Ziegmann G. (2007). Optimization of the alkali treatment process of date palm fibres for polymeric composites. Compos. Interfaces.

[18] Kong I., Tshai K.Y., Hoque M.E. (2016). Manufacturing of Natural Fibre Reinforced Polymer Composites.

[19] Mukaffa H., Asrofi M., Sujito, Asnawi, Hermawan Y., Sumarji, Qoryah R.D.H., Sapuan S.M., Ilyas R.A., Atiqah A. (2022). Effect of alkali treatment of piper betle fiber on tensile properties as biocomposite based polylactic acid: Solvent cast-film method. Mater. Today Proc..

[20] Martin O., Ave L. (2001). Poly(lactic acid): Plasticization and properties of biodegradable multiphase systems. Polymer.

[21] Dufresne A. (2006). Comparing the mechanical properties of high performances polymer nanocomposites from biological sources. J. Nanosci. Nanotechnol..

[22] Samir M.A.S.A., Alloin F., Dufresne A. (2005). Review of Recent Research into Cellulosic Whiskers, Their Properties and Their Application in Nanocomposite Field. Biomacromolecules.

[23] Bras J., Viet D., Bruzzese C., Dufresne A. (2011). Correlation between stiffness of sheets prepared from cellulose whiskers and nanoparticles dimensions. Carbohydr. Polym..

[24] Vatansever E., Arslan D., Nofar M. (2019). Polylactide cellulose-based nanocomposites. Int. J. Biol. Macromol..

[25] Gerard T., Budtova T. (2012). Morphology and molten-state rheology of polylactide and polyhydroxyalkanoate blends. Eur. Polym. J..

[26] McKeown P., Jones M.D. (2020). The Chemical Recycling of PLA: A Review. Sustain. Chem..

[27] Krikorian V., Pochan D.J. (2003). Poly (L-Lactic Acid)/Layered Silicate Nanocomposite: Fabrication, Characterization, and Properties. Chem. Mater..

[28] Signori F., Coltelli M.-B., Bronco S. (2009). Thermal degradation of poly (lactic acid) (PLA) and poly (butylene adipate-co-terephthalate) (PBAT) and their blends upon melt processing. Polym. Degrad. Stab..

[29] Metzner A.B. (1985). Rheology of Suspensions in Polymeric Liquids. J. Rheol..

[30] Al-Itry R., Lamnawar K., Maazouz A. (2012). Improvement of thermal stability, rheological and mechanical properties of PLA, PBAT and their blends by reactive extrusion with functionalized epoxy. Polym. Degrad. Stab..

[31] Nicolau A., Pop M.A., Coșereanu C. (2022). 3D Printing Application in Wood Furniture. Materials.

[32] Liao Y., Liu C., Coppola B., Barra G., Maio L.D., Incarnato L., Lafdi K. (2019). Effect of Porosity and Crystallinity on 3D Printed PLA Properties. Polymer.

[33] Figueroa-Velarde V., Diaz-Vidal T., Cisneros-López E.O., Robledo-Ortiz J.R., López-Naranjo E.J., Ortega-Gudiño P., Rosales-Rivera L.C. (2021). Mechanical and Physicochemical Properties of 3D-Printed Agave Fibers/Poly(lactic) Acid Biocomposites. Materials.

[34] Agaliotis E.M., Ake-Concha B.D., May-Pat A., Morales-Arias J.P., Bernal C., Valadez-Gonzalez A., Herrera-Franco P.J., Proust G., Koh-Dzul J.F., Carrillo J.G. (2022). Tensile Behavior of 3D Printed Polylactic Acid (PLA) Based Composites Reinforced with Natural Fiber. Polymers.

[35] Tao Y., Kong F., Li Z., Zhang J., Zhao X., Yin Q., Xing D., Li P. (2021). A review on voids of 3D printed parts by fused filament fabrication. J. Mater. Res. Technol..

[36] Bochnia J., Blasiak M., Kozior T. (2021). A Comparative Study of the Mechanical Properties of FDM 3D Prints Made of PLA and Carbon Fiber-Reinforced PLA for Thin-Walled Applications. Materials.

[37] Peng X., Zhang M., Guo Z., Sang L., Hou W. (2020). Investigation of processing parameters on tensile performance for FDM-printed carbon fiber reinforced polyamide 6 composites. Compos. Commun..

[38] Li X., Qu P., Kong H., Lei Y., Guo A., Wang S., Wan Y., Takahashi J. (2024). Enhanced mechanical properties of sandwich panels via integrated 3D printing of continuous fiber face sheet and TPMS core. Thin. Walled. Struct..

[39] Li X., Qu P., Kong H., Zhu Y., Hua C., Guo A., Wang S. (2024). Multi-scale numerical analysis of damage modes in 3D stitched composites. Int. J. Mech. Sci..

