# Supplementary material for: Biowastes as Reinforcements for Sustainable PLA-Biobased Composites Designed for 3D Printing Applications: Structure–Rheology–Process–Properties Relationships
Source: Polymers (Basel). 2025 Dec 31;18(1):128. doi: 10.3390/polym18010128 (PMC12787471; doi:10.3390/polym18010128)
Supplement: Supplementary file 1 [file polymers-18-00128-s001.zip › polymers-4039699-supplementary.pdf]

Supplementary Materials

The fiber size distribution of the treated SCB is presented in Figure S1.

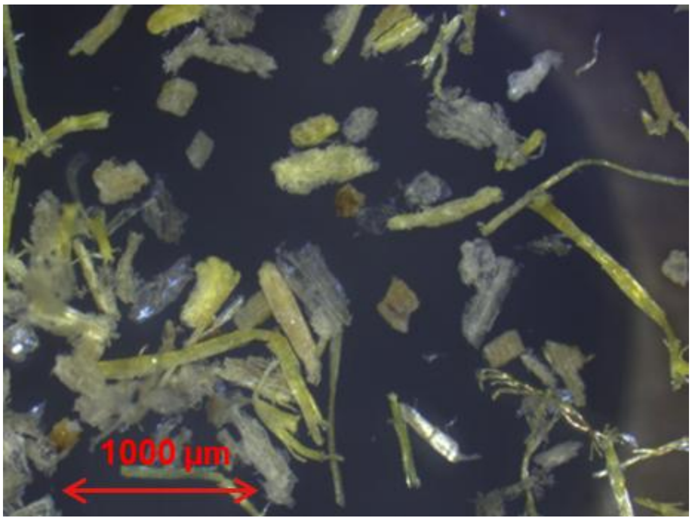

Figure S1. Image of fiber distribution by optical microscope

A1. Fourier Transformation Infrared Spectroscopy

FTIR analysis was conducted to confirm and compare the composition of the fibers before and after alkali treatment and the IR spectra can be seen in Figure S2. The aim of this part of the study was to highlight the changes in the fibers' composition following the alkali treatment. The characteristic peaks visible in Figure S2 were the following: a peak at 3373 cm^{-1} attributed to OH-stretching vibrations of cellulose [1], indicating the presence of hydroxyl groups; a peak at 2913 cm^{-1} corresponding to CH stretching [2], further confirming the cellulose structure; a distinct peak at 1734 cm^{-1} reflecting the carbonyl (C=O) stretching vibrations associated with hemicelluloses and lignin [3]; and a peak at 1640 cm^{-1} corresponding to the bending vibration of absorbed water, signifying moisture content [4]. Moreover, the aromatic ring C=C stretching vibration in lignin was marked by the peak at 1515 cm^{-1}, highlighting the presence of aromatic structures (Lara-serrano et al., 2019), the peak at 1255 cm^{-1} was an indication of the C-O stretching vibration of acetyl groups in both lignin and hemicelluloses, suggesting that these components were integral to the material [5]. Furthermore, the peak at 1047 cm^{-1}, associated with the C-O-C pyranose ring skeletal vibration, underscored the polysaccharide structure of cellulose (Stanzione et al., 2020). Finally, the peak at 895 cm^{-1} was attributed to β-glucosidic linkages between glucose units, characteristic of cellulose's molecular structure (Feng et al., 2018).

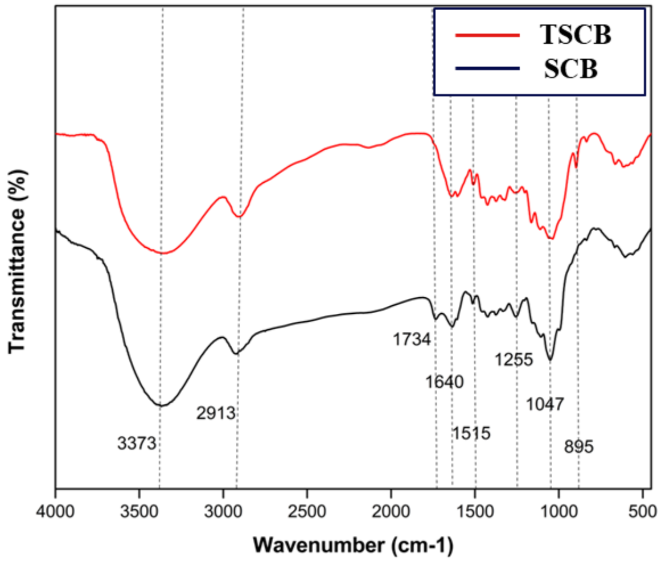

Figure S2. FTIR spectra of untreated and treated bagasse fibers

A summary of the different peaks of FTIR fibers observed is given in Table 1.

Table S1 : Identification of peak positions and corresponding chemical groups in both untreated and treated SCB fibers

Peak position (Wavenumber cm-1)	Assignments
3373	OH-stretching of cellulose [1]
2913	CH stretching of cellulose [2]
1734	Carbonyl group (C=O) stretching of hemicelluloses and lignin [3]
1640	Bending vibration of absorbed water [4]
1515	Aromatic ring (C=C) stretching vibration for lignin [6]
1255	C-O stretching vibration of acetyl groups in lignin and hemicelluloses [5]
1047	C-O-C pyranose ring skeletal vibration of cellulose [7]
895	β-glucosidic linkages between glucose units [4]

A.2. Thermal properties of bagasse fibers

A.2.1. Thermogravimetric analysis (TGA) of the fibers

During composite processing, the filler must be mixed with the polymer matrix at temperatures exceeding the melting point of the polymer. However, prolonged exposure of sugarcane bagasse fibers (TSCB) to such thermal conditions may lead to alterations in their lignocellulosic structure and composition. To better understand the thermal sensitivity of the fibers under processing conditions, a thermogravimetric analysis (TGA) was conducted to monitor their degradation behavior as a function of temperature.

Figure S3 presents the TGA and derivative mass loss (DTG) curves obtained at a heating rate of 10 °C/min. The thermal degradation of the fibers took place in several stages. The first mass loss, between 25°C and 100°C (an 8% loss), was due to the evaporation of water or moisture from the fibers [8], while the degradation of the three main constituents of natural fillers, hemicellulose, cellulose, and lignin, began at higher temperatures. According to the literature, the peak degradation of hemicelluloses typically occurs between 200°C and 350°C; in our case, it was seen between 200°C and 280°C. The second phase took place between 280°C and 350°C and corresponded to the degradation of the cellulose present in the fibers, leading to a 30% weight loss. The third phase was attributed to the decomposition of lignin between 280°C and 480°C, with a 29% mass loss. Due to its complex structure, lignin decomposes slowly, starting at around 200°C and continuing up to complete decomposition at much higher temperatures (480°C).

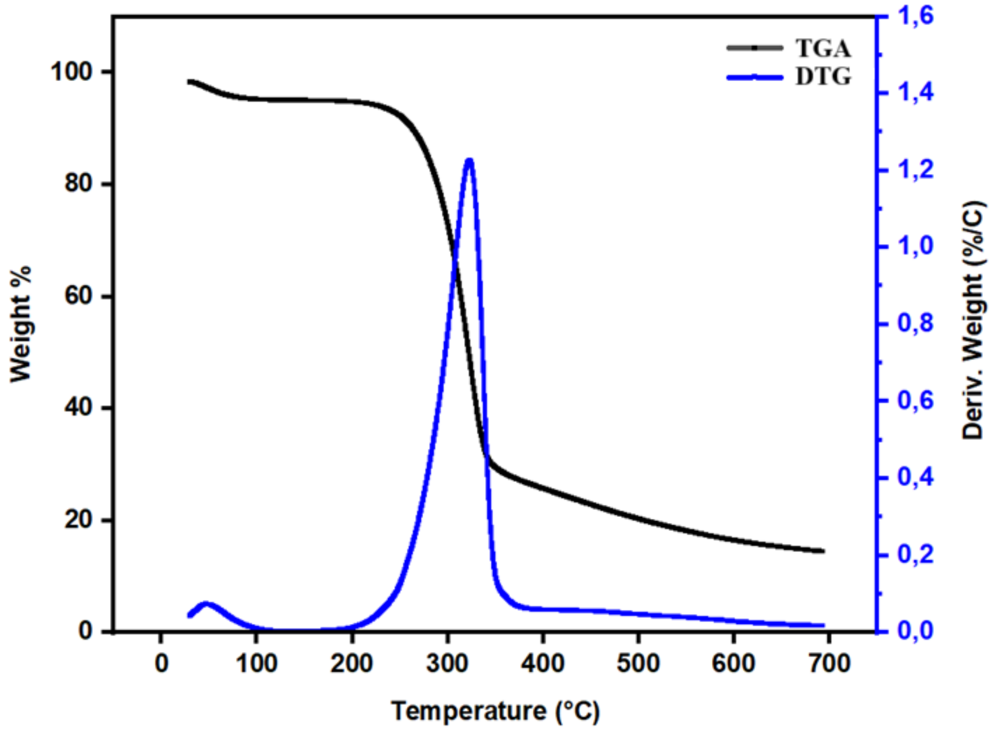

Figure S3 Typical TGA and DTG results for bagasse fibers (SCB)

A.3. Morphological properties of the bagasse fibers and bio-composites prepared by melting processing and solvent route

A.3.1 Morphological analysis of the fibers

Figure S4 displays the changes in surface morphology between untreated and alkali-treated SCB fibers. Image (4a) shows the SEM micrograph of the untreated fibers, which exhibit a surface covered with various impurities and natural residues, such as waxes and other non-cellulosic components. In addition, image (4b) shows the surface of SCB fibers after treatment with a 2% NaOH solution. The SEM micrograph reveals that the fiber surface became cleaner and more textured following the removal of surface impurities. While some traces of non-cellulosic materials may still be present, the overall surface appears rougher compared to the untreated fibers. This increased roughness is known to improve the adhesion between the fiber and the polymer matrix, as it enhances mechanical properties [9,10].In addition, this treatment allows all the chemical reactive functions that can cause the hydrolytic degradation of PLA .In summary, the SEM analysis confirms that alkali treatment helps to reduce surface contamination and improves the fiber structure for better integration into the final composite. Based on these observations, only the treated sugarcane bagasse fibers (TSCB) were selected for further composite preparation in this study. Furthermore, SEM analysis was carried out to examine the morphological characteristics of the SCB fibers in more detail. As shown in Figure S4c, the fibers exhibit a tubular and fibrillar structure with a circular cross-section. This structure reveals an organized cellular network containing small pores and a dense arrangement of microfibrils. These microfibrils appear to be held together by natural binding components. In Figure S4d, the fiber surface appears notably rough, a feature that can contribute to stronger interfacial bonding with the polymer matrix in bio-composite applications.

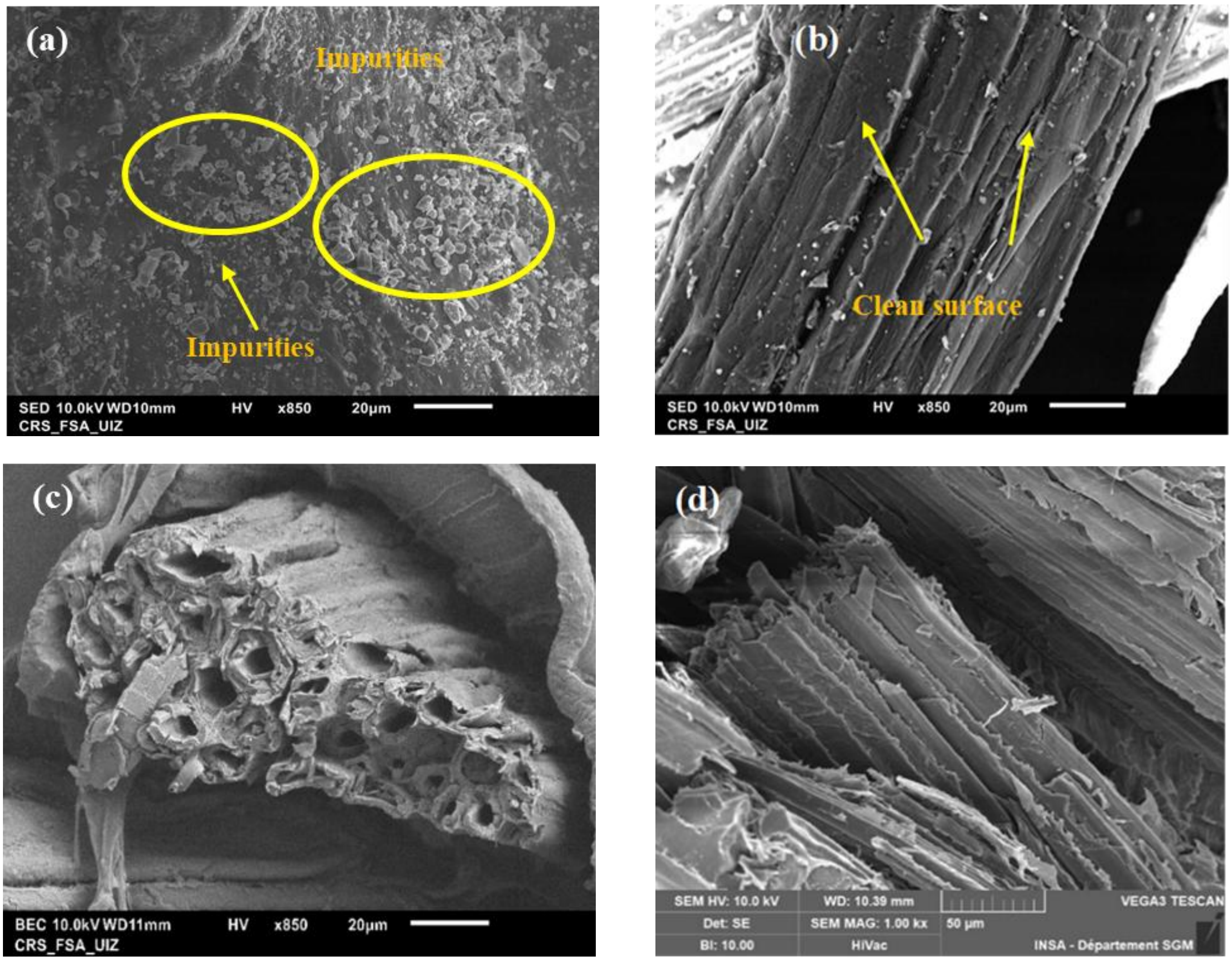

Figure S4 SEM images of (a) untreated, (b) after alkali treatment SCB fibers and (c) and (d) present SEM images showing the cross-section of an individual SCB fiber

[1] Raja K, Prabu B, Ganeshan P, Sekar VSC, Nagarajaganesh B, Prabu B, et al. Characterization Studies of Natural Cellulosic Fibers Extracted from Shwetark Stem Characterization Studies of Natural Cellulosic Fibers Extracted from Shwetark Stem 2020;0478. https://doi.org/10.1080/15440478.2019.1710650.

[2] Vijay R, Manoharan S, Arjun S, Vinod A, Singaravelu DL. Characterization of Silane-Treated and Untreated Natural Fibers from Stem of Leucas Aspera Characterization of Silane-Treated and Untreated Natural Fibers. J Nat Fibers 2020;00:1–17. https://doi.org/10.1080/15440478.2019.1710651.

[3] Njoku CE, Omotoyinbo JA, Alaneme KK, Michael O, Njoku CE, Omotoyinbo JA, et al. Characterization of Urena lobata Fibers after Alkaline Treatment for Use in Polymer Composites Characterization of Urena lobata Fibers after Alkaline Treatment for Use in Polymer Composites 2020;0478. https://doi.org/10.1080/15440478.2020.1745127.

[4] Feng Y, Cheng T, Yang W, Ma P, He H. Industrial Crops & Products Characteristics and environmentally friendly extraction of cellulose nano fi brils from sugarcane bagasse. Ind Crop Prod 2018;111:285–91. https://doi.org/10.1016/j.indcrop.2017.10.041.

[5] NagarajaGanesh B, Rekha B. Effect of mercerization on the physico-chemical properties of matured and seasoned Cocos nucifera fibers for making sustainable composites 2019.

[6] Lara-serrano M, Morales-delarosa S, Campos-mart JM, Fierro JLG. applied sciences Fractionation of Lignocellulosic Biomass by Selective Precipitation from Ionic Liquid Dissolution 2019.

[7] Stanzione M, Oliviero M, Cocca M, Errico ME, Gentile G, Avella M, et al. Tuning of polyurethane foam mechanical and thermal properties using ball- milled cellulose. Carbohydr Polym 2020;231:115772. https://doi.org/10.1016/j.carbpol.2019.115772.

[8] Sruthimol JJ, Haritha K, Warrier AS, Lal AMN, Harikrishnan MP, Rahul CJ, et al. Industrial Crops & Products Tailoring the properties of natural fibre biocomposite using chitosan and silk fibroin coatings for eco-friendly packaging. Ind Crop Prod 2025;225:120465. https://doi.org/10.1016/j.indcrop.2025.120465.

[9] Taylor P, Taha I, Steuernagel L, Ziegmann G. Optimization of the alkali treatment process of date palm fibres for polymeric composites 2013:37–41.

[10] Manimaran P, Sanjay MR, Senthamaraikannan P, Jawaid M, George R. Synthesis and characterization of cellulosic fiber from red banana peduncle as reinforcement for potential applications. J Nat Fibers 2018;00:1–13. https://doi.org/10.1080/15440478.2018.1434851.
